# Assessment of Lipid and Metabolite Changes in Obese Calf Muscle Using Multi-Echo Echo-planar Correlated Spectroscopic Imaging

**DOI:** 10.1038/s41598-017-17529-1

**Published:** 2017-12-11

**Authors:** Rajakumar Nagarajan, Catherine L. Carpenter, Cathy C. Lee, Navin Michael, Manoj K. Sarma, Raissa Souza, Edward Xu, S. Sendhil Velan, Theodore J. Hahn, Vay-Liang Go, M. Albert Thomas

**Affiliations:** 1 Radiological Sciences, University of California Los Angeles, Los Angeles, CA United States; 2 UCLA Schools of Nursing, Medicine, and Public Health, Los Angeles, CA United States; 3 Geriatric Research, Education and Clinical Center, VA Greater Los Angeles Healthcare System, Los Angeles, CA United States; 4 UCLA Department of Medicine, Los Angeles, CA United States; 5 Singapore Institute for Clinical Sciences, Singapore, Singapore; 6 Laboratory of Molecular Imaging, Singapore Bioimaging Consortium, Singapore, Singapore; 7 Departments of Physiology & Medicine, National University of, Singapore, Singapore

## Abstract

Obesity-related conditions including heart disease, stroke, and type 2 diabetes are leading causes of preventable death. Recent evidence suggests that altered myocellular lipid metabolism in obesity may lead to increased insulin resistance (IR) that predisposes to these disorders. To test the hypothesis that muscles rich in type I vs. type II muscle fibers would exhibit similar changes in intramyocellular lipid (IMCL) and extramyocellular lipid (EMCL) content in obesity, we utilized a new four-dimensional multi echo echo-planar correlated spectroscopic imaging technique that allows separate determination of IMCL and EMCL content in individual calf muscles in obese vs. normal healthy human subjects. Calf muscles were scanned in 32 obese and 11 healthy subjects using a 3T MRI/MRS scanner, and IR in the obese subjects was documented by glucose tolerance testing. In obese subjects, elevation of both IMCL and EMCL content was observed in the gastrocnemius and tibialis anterior muscles (with mixed type I and II fiber content), while a significant increase in only IMCL content (+48%, p < 0.001) was observed in the soleus muscle (predominantly type I fibers). These observations indicate unexpected differences in changes in myolipid metabolism in type I vs. type II rich muscle regions in obesity, perhaps related to IR, and warrant further investigation.

## Introduction

Obesity is associated with an increased risk of premature death^1^ and represents a rapidly-growing health problem that is reaching epidemic proportions worldwide^2^. Obesity significantly increases the risk of developing type 2 diabetes mellitus, hypertension, coronary heart disease, cancer, and stroke^3^. Weight gain and increased dietary fat intake, or production of endogenous fat from excess glucose consumption, result in the accumulation of fatty acids in liver and muscle^4^. Moreover, increased accumulation of fat in muscle has been shown to be associated with insulin resistance, dyslipidemia, and cardiometabolic disease^5^. The measurement of lipids in muscle by *in vivo* proton (^1^H) magnetic resonance spectroscopy (MRS) is complex because of the presence of two pools of lipids in skeletal muscle, intramyocellular lipids (IMCL) and extramyocellular lipids (EMCL)^6,7^. IMCL represents the presence of lipid molecules within the skeletal muscle cell, while EMCL consists of lipid molecules outside the cell. These two pools have been thought to be kinetically different, in that EMCL has been postulated to turn over slowly and serve as a long-term fat depot, whereas IMCL is thought to be in more dynamic and rapid equilibrium with substrate utilization and supply. However, these postulated differences remain to be confirmed. Increased IMCL levels may result from increased cellular uptake of fatty acids, increased lipogenesis, or reduced fatty acid oxidation. As a possible explanation for observed ectopic lipid accumulation in obese humans it has been postulated that chronic obesity is associated with excess lipid influx into muscle^8^, whereas in lean subjects who become obese, inherited or acquired impairment in mitochondrial fatty acid oxidation may play an important role in the development of insulin resistance. The measurement of IMCL by ^1^H -MRS has become of interest in the study of altered lipid metabolism in human skeletal muscle^6,7,9–14^, since it has been reported that IMCL is negatively correlated with insulin sensitivity in sedentary and diabetic volunteers^15,16^. Skeletal muscle compartments contain both oxidative and glycolytic fiber type, and there is currently considerable interest in defining the fiber type dependent changes in skeletal muscle metabolic properties that occur in obese and diabetic subjects. In human calf muscle, the soleus, tibialis anterior and gastrocnemius muscle compartments differ in terms of predominant muscle fiber type, with type I (slow twitch) fibers having higher IMCL content relative to the type II (fast twitch) muscle fibers.

Single voxel (SV) based one-dimensional (1D) MR spectroscopy of human skeletal muscle has enabled partial biochemical characterization of soleus, tibialis and other muscle regions using point resolved spectroscopy (PRESS) and stimulated echo acquisition mode (STEAM) spectroscopic sequences^17–20^. The MRS investigation of IMCL in humans has been performed most frequently using SV spectroscopy^15,16,21^ or analysis of a few selected voxels from magnetic resonance spectroscopic imaging (MRSI) data^12,13^. The lack of comprehensive MRSI studies in this area is mainly due to issues related to strong interference from lipid signals in the surrounding subcutaneous or interstitial fat and bone marrow (i.e., EMCL), which leads to contamination of the spectra of interest due to signal bleeding at low MRSI resolution. This primarily affects the quantitation of IMCL but can also complicate other metabolite identification and quantitation. Hence, the challenge has been to develop appropriate, more spatially efficient imaging techniques.

Although spectral resolution is currently a major concern, recent reports have indicated that both MRSI and SV spectroscopy have acceptable reproducibility and sensitivity for IMCL detection, although MRSI has better reliability and is more flexible in the quantitation of IMCL differences, whereas SV MRS can be acquired within a shorter scan time per voxel^22,23^. To date, significant apparent differences in IMCL content between certain muscles have been reported using MRSI techniques, with highest values reported in soleus and lower values in the tibialis anterior, tibialis posterior, and gastrocnemius muscles. In addition to these differences between muscles, significant inter-subject differences have been reported in both IMCL content and distribution over various muscles^22^. Li *et al*.^24^ have reported that two-dimensional (2D) MRSI showed improved reproducibility for IMCL quantitation as compared to SV MRS at 3T.

One-dimensional MR spectroscopic techniques are hindered by overlapping spectral resonances that can complicate metabolite identification and quantitation. However, in calf muscle, two-dimensional MRS techniques have been demonstrated to minimize the problem of spectral overlap by spreading resonances into a second (indirect) dimension, thereby improving the dispersion of spectral resonances^25,26^. In addition to estimating the IMCL and EMCL content in a specific compartment, the improved dispersion of resonances in the 2D MR spectrum, also allows us to derive additional measures of fatty acid composition like the unsaturation index. The unsaturation index gives a measure of methylene-interrupted-double bonds (present in polyunsaturated fatty acids) relative to the total double bonds (present in poly and monounsaturated fatty acids). Previously, using a single voxel based approach, we have demonstrated that unsaturation index of the skeletal muscle lipid pools is reduced in overweight and obese subjects compared to lean subjects^27,28^. In addition to the lipid peaks, the muscle MR spectrum can also be used to estimate levels of trimethylamine (TMA), which is known to be altered in obese and diabetic states^29^.

To investigate the potential utility of the MRSI technique in assessing obesity-related metabolite parameters in muscle, and begin to more accurately define changes in IMCL and EMCL in obesity, we used the recently implemented four dimensional (4D) multi-echo echo planar correlated spectroscopic imaging (ME-EPCOSI) technique^30^ to quantify lipids and metabolites in the soleus, tibialis anterior and gastrocnemius of calf muscles of obese and normal healthy subjects. The present study utilized 4D ME-EPCOSI to rapidly characterize the changes in IMCL, EMCL and lipid unsaturation levels within each calf muscle compartment in obese vs. non-obese healthy subjects. In a subset of the obese subjects, we also evaluated the association between the above measurements and insulin sensitivity.

## Materials and Methods

### Human Subjects

The objective of our study was to examine potential changes in lipid myocellular distribution in the calf muscle region of obese individuals. In addition to obese subjects, we also recruited normal healthy subjects to serve as reference standards for the scanning protocols. The ME-EPCOSI sequence was tested in the calf muscle of 32 obese subjects (mean age 28 years old) and eleven normal healthy subjects (mean age 32 years old). The mean and standard deviation (SD) of body mass indexes (BMI) for the obese and healthy subjects were 38.9 + 5.1 and 23.6 + 1.8 kg/m^2^ respectively. The eligibility criteria for this study were as follows: age between 18–35 years old, not in an exercise or weight management program within the past 6 months, non-smoker, not diabetic, no history of gastric surgery for obesity (gastric bypass, lapband procedure), and, if female, not pregnant. Table 1 describes the demographic distribution of the obese and healthy subjects. Our subject recruitment procedures and study protocol were Health Insurance the Portability and Accountability Act (HIPAA) compliant and performed with the approval of UCLA Institutional Review Board. Experiments were carried out in accordance with approved guidelines, and informed consent was obtained from all subjects.

**Table 1:** Demographics of obese and healthy subjects.

Characteristics	Obese Subjects (*n* = 32)	Healthy Subjects (*n* = 11)
Male	15 (53.1%)	8 (72.7%)
Female	17 (46.9%)	3 (27.3%)
Asian	2 (6.2%)	7 (63.6%)
Black	4 (12.5%)	1 (9.1%)
White	19 (59.4%)	3 (27.3%)
White/Hispanic	7 (21.9%)	None
Mean Age (Years)	28	32
<20 years	2 (6.3%)	None
20–24 years	9 (28.1%)	None
25–29 years	9 (28.1%)	5 (45.5%)
≥30 years	12 (37.5)	6 (54.5)
BMI (kg/m^2^)	38.9 ± 5.1	23.6 ± 1.8

### Oral Glucose Tolerance Test

To assess insulin sensitivity, an oral glucose tolerance test was performed in a subset of 19 obese subjects. During the baseline assessment period, using a standardized protocol participants were instructed to undergo 12 hours of fasting (overnight) and to not engage in vigorous exercise for 24 hours prior to this test. Venous blood samples were obtained at baseline and every 30 minutes (30, 60, 90 and 120) after consuming 75 g of anhydrous glucose dissolved in water. Blood plasma samples were frozen until thawed to undergo batch measurement of insulin by radioimmunoassay^31^ and glucose levels by the glucose oxidase method (YSI 2300 glucose analyzer, Yellow Springs, OH). The glucose and insulin values were used to estimate insulin sensitivity using the Matsuda index^32^.

### 4D ME-EPCOSI Acquisition

Subjects were placed in the MRI scanner feet first in the supine position. The right calf of each subject was placed flat on a 15 channel ‘receive’ and a single channel ‘transmit’ knee coil, with the largest diameter of the calf muscle positioned in the middle of the coil. The axial, coronal and sagittal images were used to select the position of the volume of interest (VOI, also known as voxel), in the tibialis anterior, soleus and gastrocnemius muscles of the calf. Voxel positions were selected so as to avoid interference from vascular structures and gross adipose tissue deposits, and to ensure that the muscle fibers were consistently oriented along the magnetic field. All scans were performed on a Siemens (Siemens Medical Solutions, Erlangen, Germany) 3T Trio-TIM MRI scanner running on the VB17a platform. Anatomical MRI studies included four different MRI scans: a three-plane localizer MRI (Repetition time/Echo time (TR/TE) = 14/5ms, field of view (FOV) = 45 × 45 cm^2^, a 320 × 160 data matrix, one average) and a T_1_-weighted (T_1_W) axial, coronal and sagittal spin-echo MRI (TR/TE = 600/2.7 ms, FOV = 16 × 16 cm^2^, 320 × 256 data matrix, one average). We also acquired T_1_W MR Images in sagittal and transverse orientations to localize the 4D ME-EPCOSI. The parameters for the MEEPCOSI acquisition were: TR/TE = 1.5 s/30 ms, one average, 16 phase encoding steps, FOV = 16 × 16 cm^2^, and 256 complex points with an F_2_ bandwidth of 1190 Hz. For the second dimension (F_1_), 50 increments with bandwidths of 1250 Hz were used. The individual voxel volume extractable in calf was 2 ml. Manual B_0_ shimming was performed to minimize B_0_ inhomogeneity over the localized VOI and the full-width-at-maximum (FWHM) of the unsuppressed water was typically 16 Hz. A non-water-suppressed (NWS) scan was acquired using 256 t_2_ points, one average with only one t_1_ (Δt_1_ = 0) and 16 × 16 spatial encoding as a reference to correct for eddy current distortions created by the echo planar spectroscopic imaging (EPSI) readout. The second reference acquisition took 30 seconds including 4 dummy scans to reach the steady state equilibrium.

The 4D ME-EPCOSI data were processed using MATLAB codes through a sequence of steps comprising scaling, spatial reordering, phase correction, resolving averages and oversampling^30^. Fast Fourier Transforms (FFT) were applied twice to the filtered data, first along the k_x_ and k_y_ dimensions to yield the spatial dimensions x (left to right) and y (anterior to posterior), respectively. FFTs were also applied along the time dimensions t_1_ and t_2_ to yield the frequency dimensions F_1_ and F_2_, respectively. The final output comprised a 4D matrix set in the format S (F_2_, x, y, F_1_). All of the 4D ME-EPCOSI data from soleus, tibialis anterior and gastrocnemius muscles were processed using a home-built graphic user interface (GUI) based MATLAB program. Figures illustrating the implemented GUI are included in the Supplemental Material to understand the types of lipids and the range of chemical shift used in the GUI automated processing.

### 2D MRS peak analysis

The 2D diagonal and cross peaks were defined as (1) unsaturated fatty acids, Unsat_CH = CH (5.4 ppm, 5.4 ppm); (2) intramyocellular lipids: IMCL1 and IMCL2 ((5.3 ppm, 2.7 ppm) and (5.3 ppm, 2.0 ppm); (3) extramyocellular lipids: EMCL1 and EMCL2 (5.45 ppm, 2.85 ppm) and (5.45 ppm, 2.15 ppm); (4) choline, Ch_d (3.2 ppm, 3.2 ppm), and (5) creatine, Cr_d (3.0 ppm, 3.0 ppm). The acquired 4D ME-EPCOSI data was processed using a MATLAB based GUI, which was developed in-house. Absolute volumes of the above mentioned peaks were calculated for different 2D spectra recorded in the soleus, tibialis anterior and gastrocnemius muscles regions of all subjects using peak integration. In MRS, concentrations are usually expressed in relative terms, with reference to an internal or external concentration reference. Since total muscle creatine content does not change with obesity, it was used as an internal reference for the other peaks in the muscle spectrum. The IMCL, EMCL and choline peak ratios were calculated by dividing the absolute peak volume of the diagonal peaks by that of the Cr_d peak. The unsaturation indices (UI) of IMCL and EMCL were estimated using the ratio of the cross-peaks, IMCL1/IMCL2 and EMCL1/EMCL2, respectively^26–28^.

### Statistical Analysis

Data from the IMCL, EMCL and choline (Cho) to creatine ratios (IMCL/Cr, EMCL/Cr, and Cho/Cr), and lipid unsaturation indices for each muscle (soleus, tibialis anterior and gastrocnemius) were read into SAS 9.4™ (SAS Institute, 2012; Cary, NC). For comparisons of obese vs. normal healthy subject values within different muscle groups, two-tailed unpaired t-tests were performed with p < 0.05 assumed to be statistically significant. In the data for both obese and normal healthy subjects, Pearson’s correlations were performed to examine relationships between the metabolite ratios for various muscle compartments (soleus, tibialis anterior and gastrocnemius). The presence of outliers in the insulin sensitivity measurements was assessed using a boxplot. One subject, whose Matsuda index exceeded the third quartile by more than 1.5 times the interquartile range, was excluded from the analysis. The degree of association of the Matsuda index with the IMCL-to-creatine ratios, EMCL-to-creatine ratios and IMCL and EMCL unsaturation indices in the soleus, gastrocnemius and tibialis anterior muscle compartments were assessed using the Pearson correlation coefficient. The level of statistical significance was set at p < 0.05 for all analyses.

## Results

**Figure 1:**
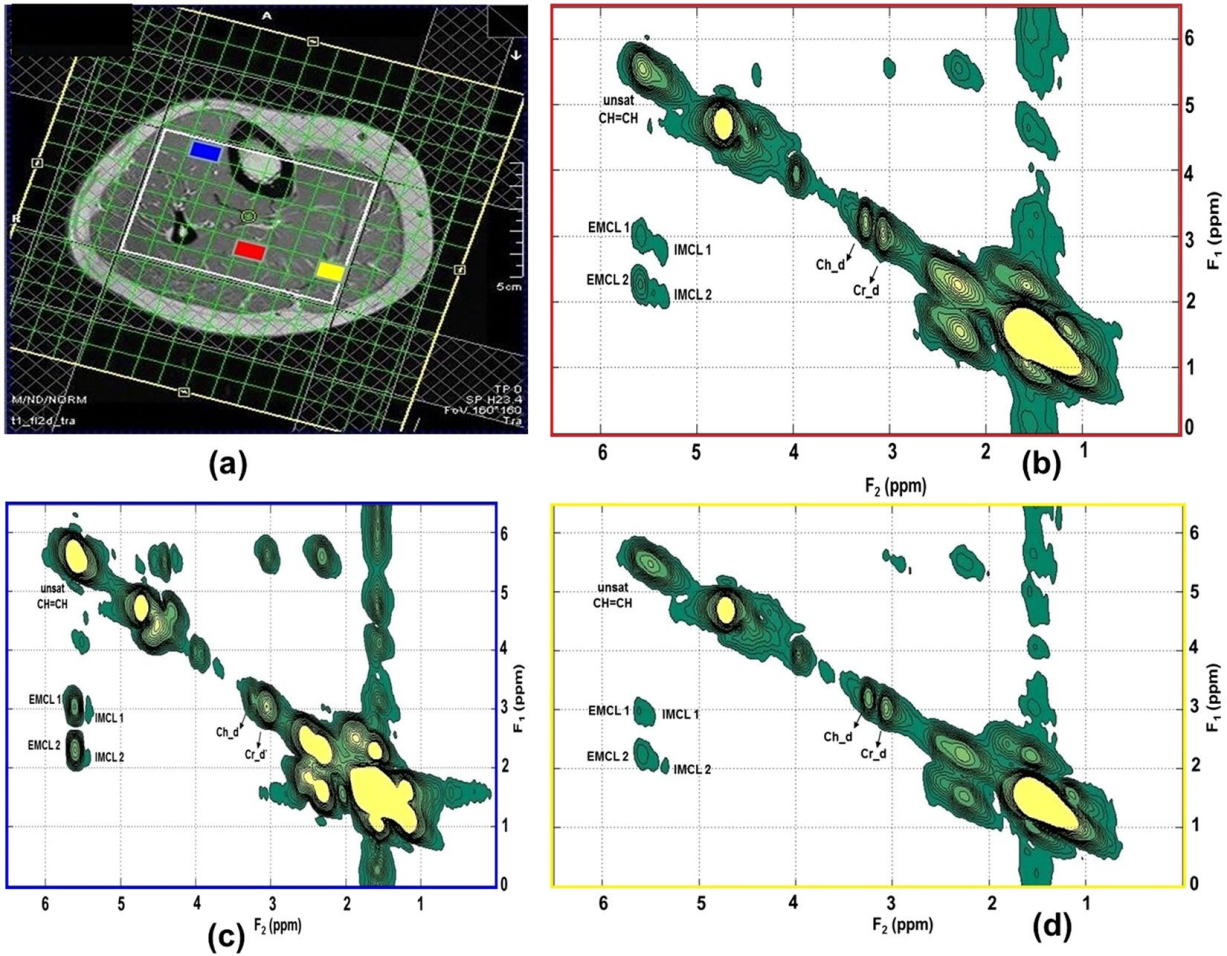
MRI and 4D ME-EPCOSI voxel placement (**a**) in a 25 years old obese subject with extracted soleus (**b**) tibialis anterior (**c**) and gastrocnemius muscle (**d**) spectra.

**Figure 2:**
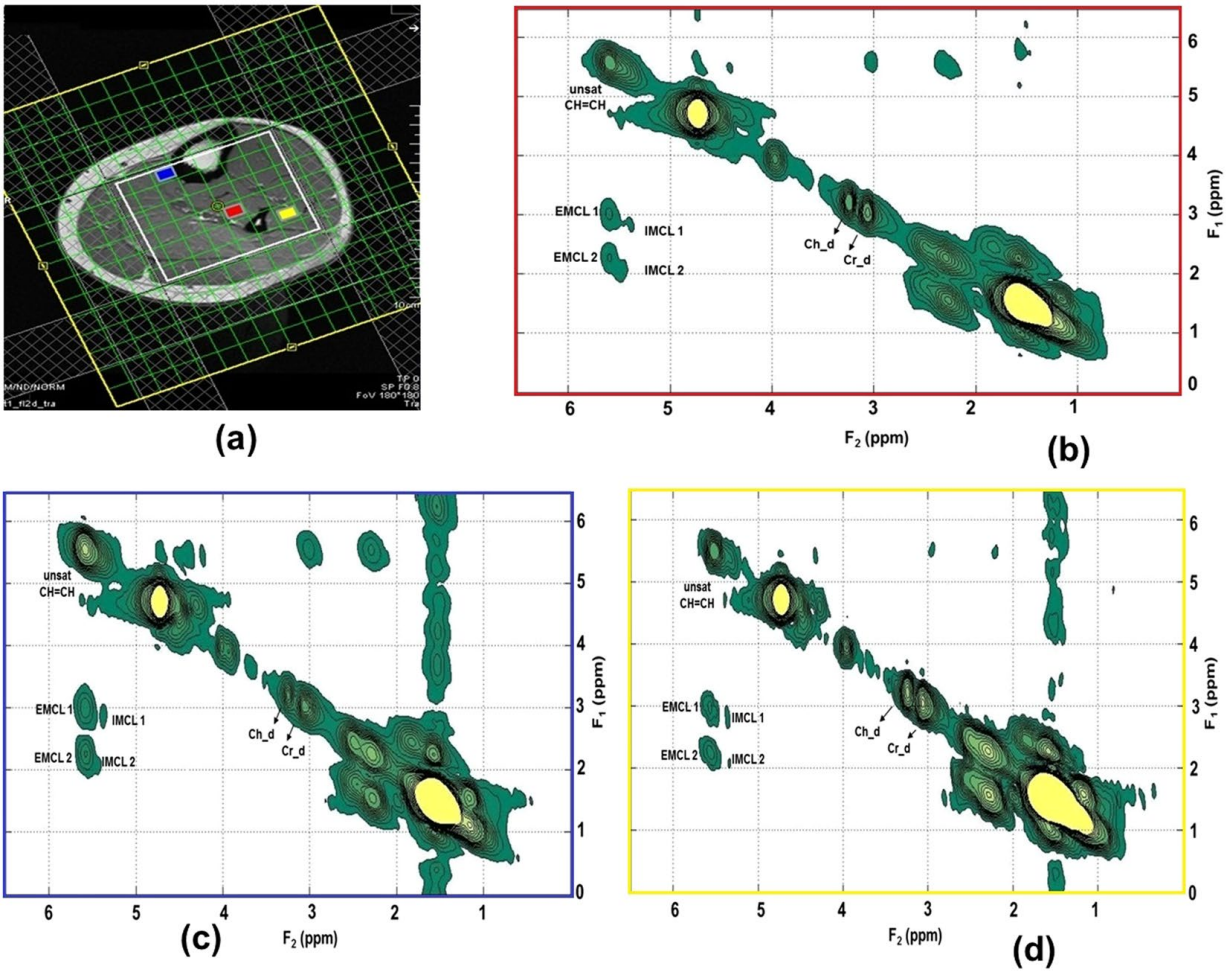
MRI and 4D ME-EPCOSI voxel placement (**a**) in a 27 years old normal healthy subject with extracted soleus (**b**) tibialis anterior (**c**) and gastrocnemius muscle (**d**) spectra.

**Figure 3:**
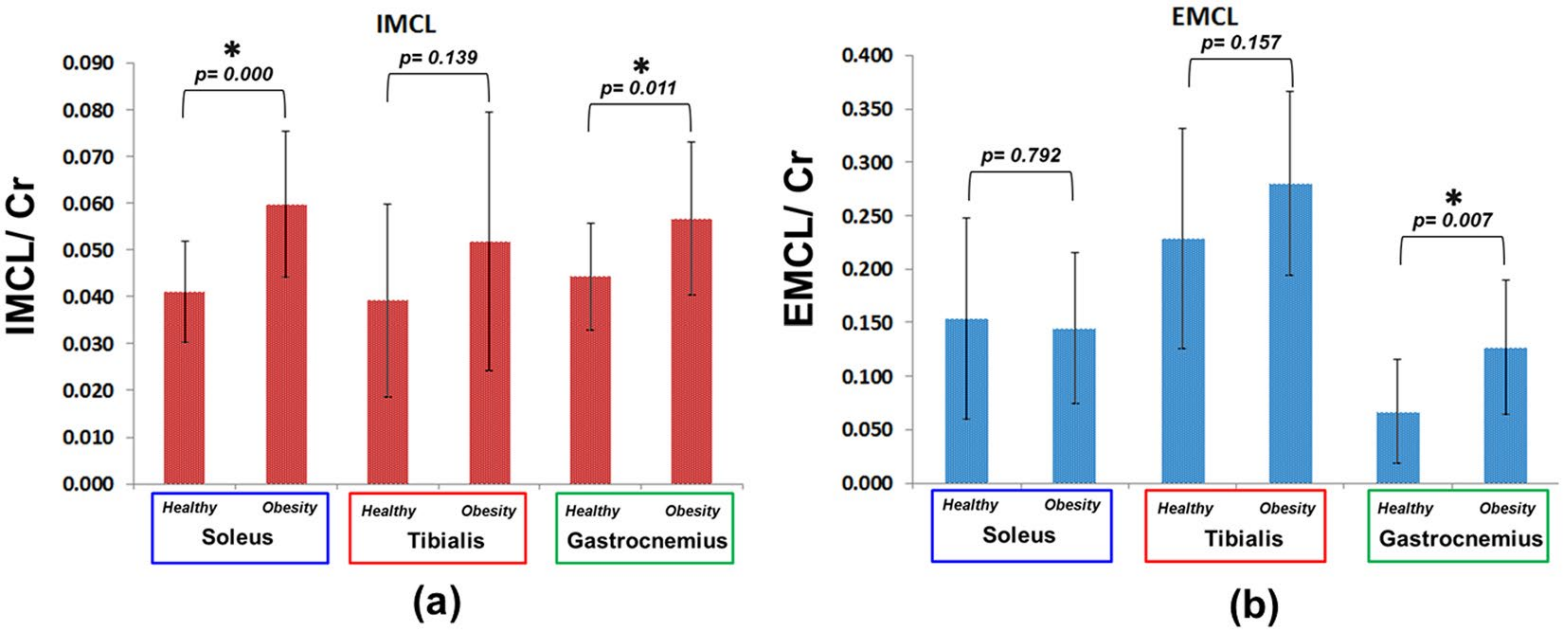
IMCL and EMCL creatine ratios of soleus, tibialis anterior and gastrocnemius muscles of obese and normal healthy subjects.

**Figure 4:**
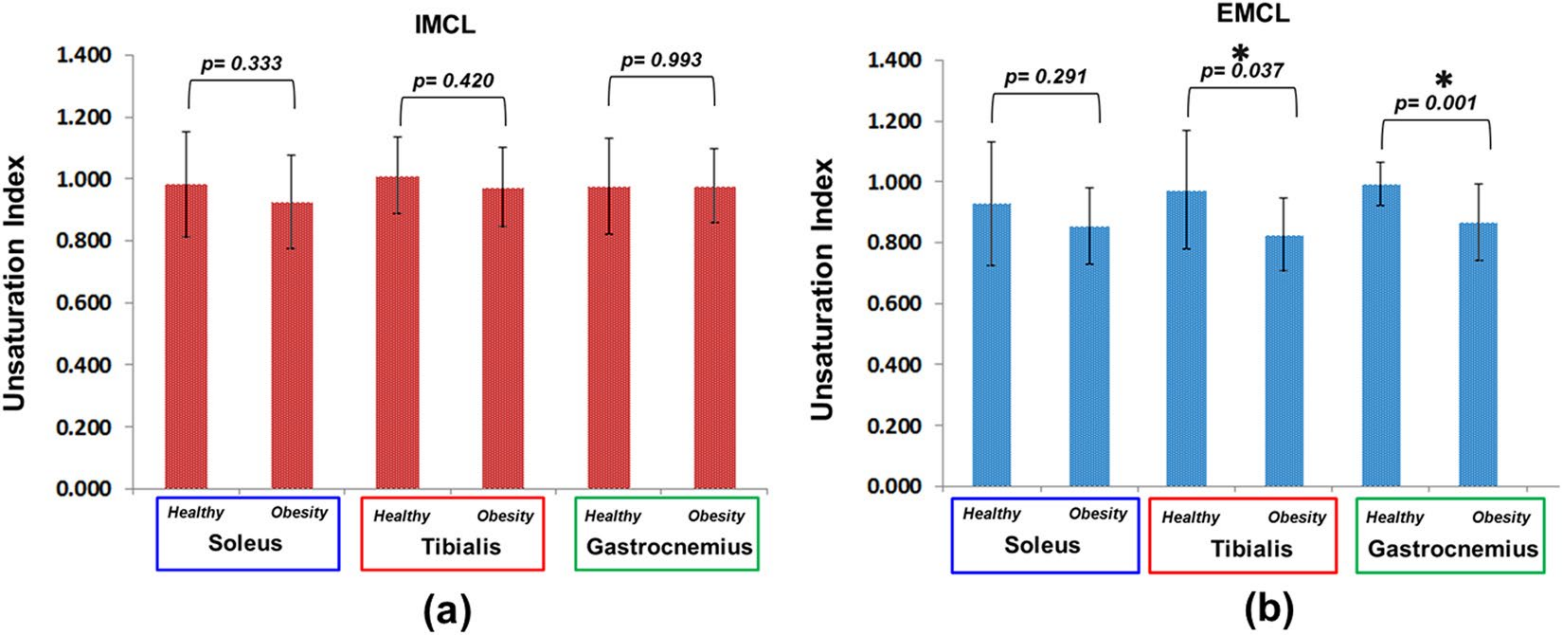
Unsaturation indices of soleus, tibialis anterior and gastrocnemius muscles of obese and normal healthy subjects.

Figures 1 and 2 show the MRI and 4D ME-EPCOSI voxel placement in a 25-year-old obese and 27-year-old normal healthy subject with 2D spectra extracted from soleus, tibialis anterior and gastrocnemius muscles. Increased IMCL and EMCL were observed in all three highlighted muscle locations (soleus, tibialis anterior and gastrocnemius). Figure 3a and b illustrate the mean values of the IMCL/Cr and EMCL/Cr ratios in the soleus, tibialis anterior and gastrocnemius muscles in obese vs. normal healthy subjects. As shown in Figure 3a, a significant increase in IMCL/Cr was observed in the soleus muscle of obese subjects relative to healthy subjects (+48%, P < 0.001), while there was no significant change seen in EMCL/Cr in the soleus in obese subjects. In contrast, a significant increase in both IMCL/Cr and EML/Cr was observed in the gastrocnemius and there was also a non-significant trend toward an increase in both IMCL/Cr and EMCL/Cr in the tibialis anterior. Of note, previous histologic studies have shown that the soleus is the calf muscle compartment with the highest proportion of type I (slow) muscle fibers, with type I fiber content being significantly lower in the gastrocnemius. The lipid unsaturation indices (UIs) of soleus, tibialis anterior and gastrocnemius muscles of obese and healthy subject are shown in Fig. 4a and b.

As shown in Fig. 4a, there were no significant differences observed in the IMCL UI levels in obese vs. healthy subjects in any of the three calf muscle groups. In contrast however, there was a significant decrease in the EMCL UI in obese subjects observed in both the tibialis and gastrocnemius muscles, with a similar non-significant trend observed in the soleus.

**Figure 5:**
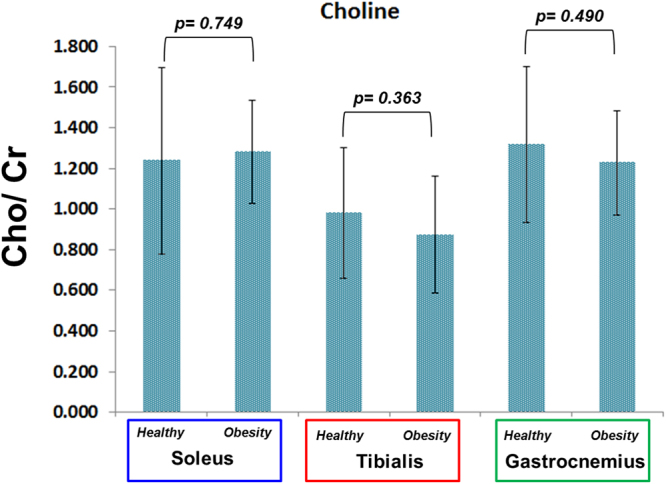
Choline to creatine ratios of soleus, tibialis anterior and gastrocnemius muscles of obese and normal healthy subjects.

The Cho/Cr ratios in the soleus, tibialis anterior and gastrocnemius muscles of obese and healthy subjects are shown in Fig. 5. While there were differences in Cho/Cr levels among the three muscle groups in healthy subjects, there were no observed differences between the obese and normal healthy subjects.

**Figure 6:**
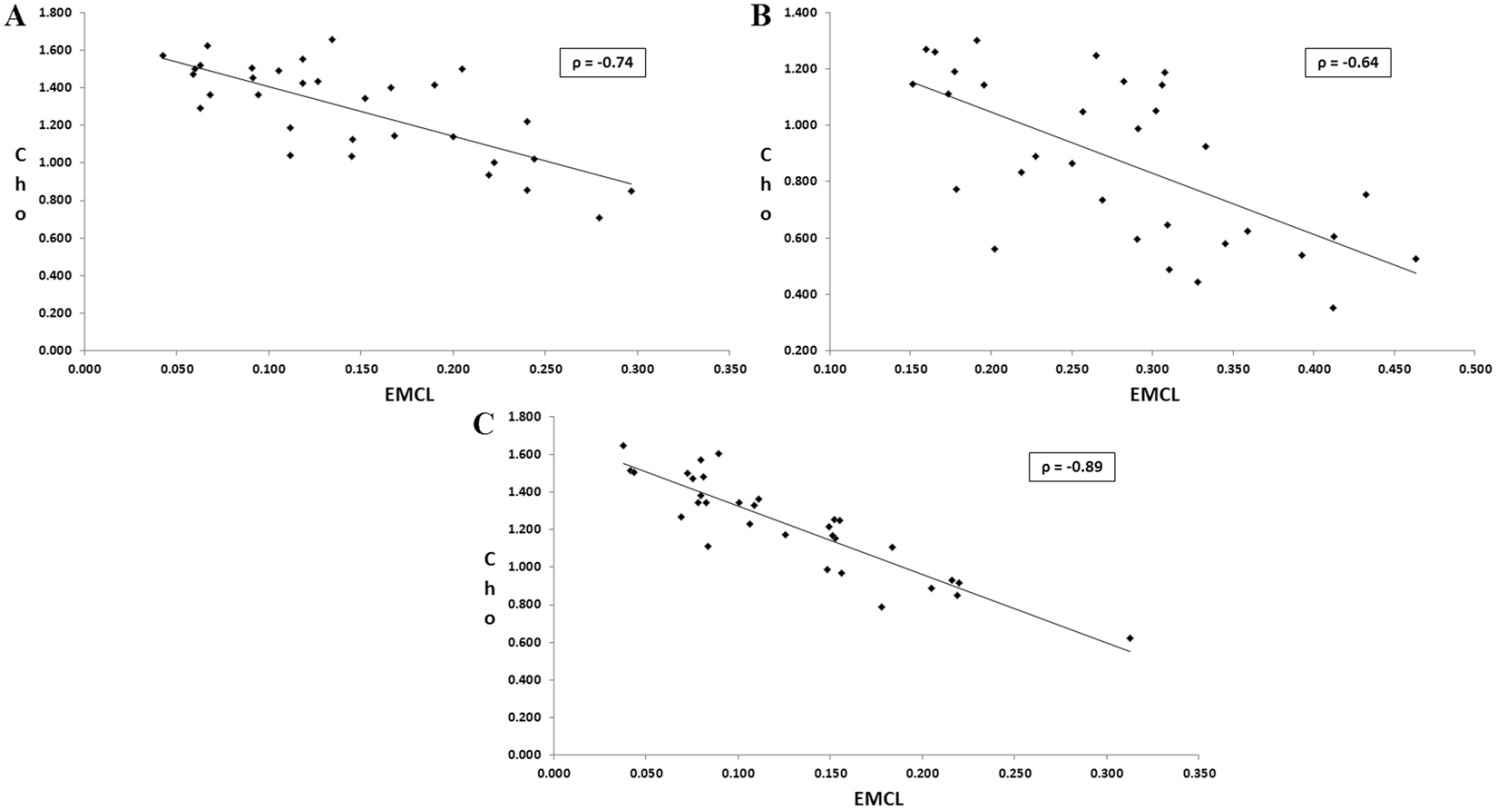
Correlations between choline and extramyocellular lipids in the (**A**) soleus, (**B**) tibialis anterior. and (**C**) gastrocnemius muscle regions of obese adults.

There was a significant increase in IMCL/Cr in the soleus and gastrocnemius muscles in obese as compared to normal healthy subjects, with a similar non-significant trend. Also, a significant increase of EMCL/Cr was observed in the gastrocnemius muscles of obese subjects. Significant decrease of the EMCL unsaturation index was observed in tibialis anterior and gastrocnemius muscle in obese subjects. In addition, a non-significant trend for decreased Cho/Cr was observed in the tibialis anterior and gastrocnemius muscles of obese individuals. Table 2 shows the significant correlations between metabolite ratios and various muscles in the normal healthy group. In the obese group, negative correlations appeared between EMCL and Cho in the soleus muscle. To further examine the patterns observed between EMCL and Cho in the obese group, we plotted individual values for both EMCL and Cho as shown in Fig. 6. Non-significant correlations were observed between Cho and IMCL in all three muscle regions. As shown in Table 2, in the healthy group, negative correlations appeared between EMCL and Cho levels and positive correlation observed in IMCL and Cho ratios in the soleus. We observed negative correlations between EMCL and Cho levels, as well as between the IMCL and EMCL levels, in the tibialis anterior. A positive correlation was observed between IMCL and EMCL group in the gastrocnemius. Table 3 indicates the associations of the Matsuda index with IMCL creatine ratios, and IMCL unsaturation indices in the three muscle groups, evaluated in 16 obese subjects. Table 4 indicates the corresponding associations of the Matsuda index with EMCL/Cr ratios and EMCL unsaturation indices. Weak to moderate negative associations were observed between Matsuda index and IMCL/Cr in the soleus and gastrocnemius muscles, but not the tibialis anterior muscle. Moderate to strong positive associations were observed between Matsuda index and IMCL unsaturation indices. The associations observed in the gastrocnemius compartment were stronger than the associations observed in the other muscle compartments. Only the associations of the Matsuda index with IMCL/Cr ratio and IMCL unsaturation index in the gastrocnemius compartment were statistically significant. Matsuda index was positively associated with the EMCL/Cr ratio in the soleus and gastrocnemius muscle, but negatively in the tibialis anterior muscle. A strong positive statistically significant association was observed between the Matsuda index and EMCL unsaturation index in the gastrocnemius muscle. Statistically non-significant positive associations were observed between Matsuda index and EMCL unsaturation index in the soleus and tibialis anterior muscle.

**Table 2:** Correlations between ratios of lipids and metabolites and different healthy muscles.

Healthy			
Metabolites	Location	Correlation Coefficient	p-value
EMCL *Vs* Cho	Soleus	−0.52	0.01
IMCL *Vs* Cho	Soleus	0.54	0.08
EMCL *Vs* Cho	Tibialis Anterior	−0.84	0.002
IMCL *Vs* EMCL	Tibialis Anterior	−0.58	0.07
IMCL *Vs* EMCL	Gastrocnemius	0.63	0.06

**Table 3:** Association of Matsuda index of insulin sensitivity with IMCL/Cr and IMCL unsaturation indices in different muscle regions.

Measurement	Location	Correlation Coefficients (r)	P-value
IMCL/Cr	Soleus	−0.157	0.562
	Tibialis Anterior	0.085	0.755
	Gastrocnemius	−0.462	0.071
IMCL Unsaturation Index	Soleus	0.304	0.253
	Tibialis Anterior	0.400	0.125
	Gastrocnemius	0.558	0.025

**Table 4:** Association of Matsuda index of insulin sensitivity with EMCL/Cr and EMCL unsaturation indices in different muscle regions.

Measurement	Location	Correlation Coefficients (r)	P-value
EMCL/Cr	Soleus	−0.285	0.285
	Tibialis Anterior	0.332	0.210
	Gastrocnemius	−0.349	0.186
EMCL Unsaturation Index	Soleus	0.246	0.358
	Tibialis Anterior	0.123	0.651
	Gastrocnemius	0.513	0.042

## Discussion

Our study indicates regional differences in IMCL and EMCL accumulation and unsaturation with increased obesity in the calf muscles. Interestingly, increased IMCL content by direct muscle biopsy measurement has previously been described as an early marker of the development of insulin resistance^33,34^. A study conducted in normoglycemic individuals that directly assessed muscle triglyceride content using histological and biochemical methods found a negative association between increased triglycerides and decreased glycogen synthase activity^35^. Also, histochemically determined high IMCL content was also related to high waist-to-hip ratios and high free fatty acid plasma concentrations in the same study^35^. These results suggest that high IMCL content, possibly related to high plasma fatty acid levels, can lead to the development of insulin resistance, although the possibility cannot be excluded that insulin resistance also leads to the accumulation of IMCLs.

In addition, increased IMCL accumulation may be linked to the fat constituents of the diet. Consuming high-fat diets for varying lengths of time has been shown to increase IMCL content by 36–90%, depending on duration and initial IMCL level^36–38^. In physically inactive obese subjects, a continuous increased source of fatty acids by way of excessive food energy intake, together with a reduced capacity to oxidize fat, may lead to overall increased fat storage, specifically in skeletal muscle, thereby further impairing insulin sensitivity.

Our data indicated significantly elevated IMCL levels in the soleus and gastrocnemius muscles, of obese subjects but not in the tibialis anterior muscle. This may be partly influenced by the muscle fiber composition. IMCL is generally higher in type I fibers compare to type II fibers. Among the three muscles, the tibialis anterior muscle has the lowest type I fiber content, while the soleus muscle has the highest type 1 fiber content.

The unsaturation index as defined in this work, gives a measure of the methylene-interrupted double bonds relative to the total double bonds. Thus the unsaturation index is sensitive to changes in polyunsaturated fatty acids relative to monounsaturated fatty acids. Unlike an earlier study that analyzed IMCL unsaturation in a single voxel (27 ml) with a longer scan time >15 mins, which indicated reduced unsaturation with increased obesity, no clear changes in unsaturation were observed within the IMCL lipid pool with increased obesity in the current study^27^. A surprising finding was a significant reduction in the EMCL unsaturation index in the tibialis anterior and gastrocnemius muscles of obese subjects. It is not clear if the unsaturation index is influenced by the muscle fiber composition. Both the above muscles have relatively high type II fibers.

In the subset of obese subjects, in which the oral glucose tolerance test was performed, we found weak to moderate negative associations between Matsuda index of insulin sensitivity and IMCL/Cr ratios, with the strongest association in the gastrocnemius muscle. IMCL/Cr ratios in both tibialis anterior and soleus muscle shave been reported to be negatively associated with insulin sensitivity. Because of our relatively small samples size, we did not attempt to stratify subjects by ethnicity or gender, both of which are known to modify the association between IMCL and insulin sensitivity. This may explain the weaker associations in the tibialis anterior and soleus compartments. The association between Matsuda index and EMCL/Cr ratio was negative in the soleus and gastrocnemius muscles and positive in the tibialis anterior muscles. It is difficult to unambiguously interpret the associations of spectroscopy derived EMCL levels due to the heterogeneity of extramyocellular adipocyte infiltration. EMCL levels obtained from specific voxel, within a muscle compartment may not be representative of the EMCL levels across the entire muscle. We observed consistent positive associations between both IMCL and EMCL unsaturation indices and the Matsuda index.

The reduced EMCL unsaturation index in obese subjects and the positive association between unsaturation index and insulin sensitivity, suggests a potential link between increased oxidative degradation of polyunsaturated fatty acids and reduced unsaturation index. Obesity can independently cause increased lipid peroxidation by progressive and cumulative cell injury resulting from pressure due to expanding adipose tissue mass. Cell injury causes the release of cytokines, especially tumor necrosis factor alpha, which generates reactive oxygen species from the tissues which in turn cause lipid peroxidation^39^. Lipid peroxidation is known to be involved in a number of human pathologies including atherosclerosis and hypertension^39,40^. Polyunsaturated fatty acids, which have multiple double bonds, are more susceptible to lipid peroxidation than monounsaturated fatty acids. Reduced unsaturation index could thus be a potential marker for increased degradation of polyunsaturated fatty acids due to lipid peroxidation^26–28^. Lipid peroxidation within skeletal muscle has been linked to increased insulin resistance^41^ and can result in increased saturation of membrane phospholipids. This reduces the membrane fluidity, which in turn has been linked to increased insulin resistance^42^. This may partly explain the positive association between the Matsuda index and the unsaturation indices.

One of the dominant resonances in ^1^H MRS at 3.2 ppm (Cho) stems from the trimethyleamine (TMA) pool which includes glycerylphosphocholine (GPC), phosphocholine (PCh), free choline (Ch) and ethanolamine groups. In addition, carnitine and acetylcarnitine belong to the TMA pool, and both molecules play critical roles in muscle metabolism^43^. Increased and decreased trends in muscle TMA levels have been reported in obese subjects compared to normal healthy subjects in all three muscle groups, although in the current study, the difference was non-significant, as shown in Fig. 5. We observed a non-significant decrease in Cho in tibialis anterior and gastrocnemius muscle regions which is in agreement with our earlier work comparing diabetic and healthy subjects^30^.

Most of the earlier MR studies of muscle lipid levels were limited to single voxel or MRSI based approaches to attempt to quantitate lipid types and levels. By using 4D ME-EPCOSI, we were able to both markedly increase the spatial coverage and lipid differentiation in skeletal muscle and complete the scans in a shorter time (10 mins). Our results demonstrated higher IMCL and EMCL levels in obese compared to normal healthy subjects. We also observed a decreased lipid unsaturation index in obese subjects compared with normal healthy subjects, and positive associations between lipids unsaturation indices and insulin sensitivity. The reduced degree of unsaturation in obese subjects may result from increased lipid peroxidation^44^. Most of the prior MR based investigations of the metabolic effect of IMCL accumulation have been limited to a single voxel in either the soleus or tibialis anterior muscles. In the study, we found stronger associations of gastrocnemius muscle IMCL/Cr ratios and lipid unsaturation indices with insulin sensitivity when compared to soleus and tibialis anterior muscles. Few drawbacks of the current study include the small group of obese and normal healthy subjects and lack of the OGTT data in healthy subjects. However, a future study including a larger cohort of obese and normal healthy subjects will address these problems.

## Conclusion

The 4D ME-EPCOSI acquired data enabled quantitation of unsaturated and saturated fatty acids (and metabolites) in different calf muscle regions using IMCL ratios and unsaturation indices. The ME-EPCOSI technique has a major potential to be valuable in various clinical studies, such as diabetes and heart failure where the levels of IMCLs in various muscles can be used as markers for differences in metabolic activity, disease activity and response to therapy.

## Electronic supplementary material

Supplementary material
