# Supplementary material for: Assessment of Lipid and Metabolite Changes in Obese Calf Muscle Using Multi-Echo Echo-planar Correlated Spectroscopic Imaging
Source: Sci Rep. 2017 Dec 11;7:17338. doi: 10.1038/s41598-017-17529-1 (PMC5725420; doi:10.1038/s41598-017-17529-1)
Supplement: Supplementary file 1 — Supplementary material [file 41598_2017_17529_MOESM1_ESM.pdf]

Assessment of Lipid and Metabolite Changes in Obese Calf Muscle Using Multi-Echo Echo-planar Correlated Spectroscopic Imaging

Rajakumar Nagarajan[1], Catherine L. Carpenter[2], Cathy C Lee[3,4], Navin Michael[5], Manoj K Sarma[1], Raissa Souza[1], Edward Xu[1], S. Sendhil Velan[6,7], Theodore J. Hahn[3,4], Vay-Liang Go[4], and M. Albert Thomas[1]

[1]Radiological Sciences, University of California Los Angeles, Los Angeles, CA, United States,
[2]UCLA Schools of Nursing, Medicine, and Public Health, Los Angeles, CA, United States,
[3]Geriatric Research, Education and Clinical Center, VA Greater Los Angeles Healthcare System, Los Angeles, CA, United States,
[4]UCLA Department of Medicine, Los Angeles, CA, United States,
[5]Singapore Institute for Clinical Sciences, Singapore
[6]Laboratory of Molecular Imaging, Singapore Bioimaging Consortium
[7]Departments of Physiology & Medicine, National University of Singapore, Singapore

,

Running Title: *Fast MR Spectroscopic Imaging of Obese Calf*

Supplemental Figures about GUI

S1. Home-developed Matlab-based GUI software for post-processing 4D MEEP-COSI: The figure below shows the main window of the EPCOSI/MEEPCOSI GUI.

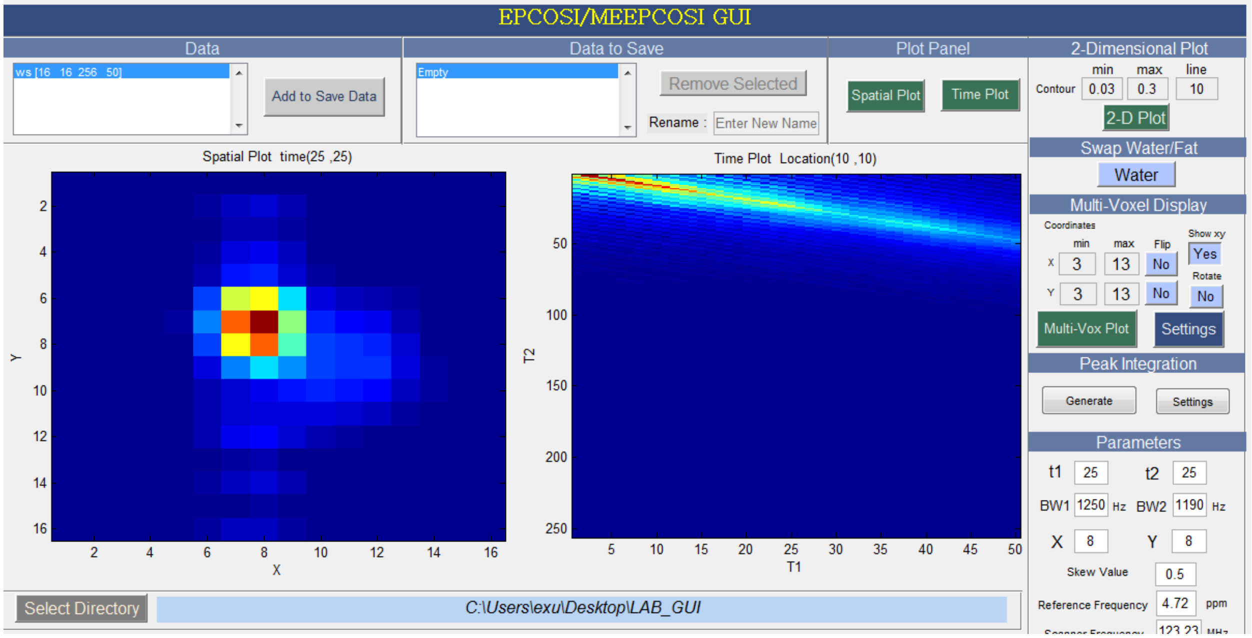

Processed outputs will appear in the Data section and can be archived using the Data to ‘Save’ commands. In addition, spatial and time plots can be generated according to the entered Parameters. 2-D contour plots for metabolite concentration can be created for any given voxel at coordinate X, Y (found in Parameters section) using the 2-Dimensional Plot option (S3-A). The Multi-Voxel Display generates 2-D contour plots for all voxels from Xmin to Xmax and Ymin to Ymax. This allows side-by-side comparison of specific peaks, which can be further refined through the Settings button (S3-B).

S2. Peak Integration window of the GUI software. The Peak Integration feature extracts metabolite concentration data based upon the 2-D plot. For each peak selected, the GUI will sum all data points within a set coordinate range representative for the given metabolite and output the values to a spreadsheet.

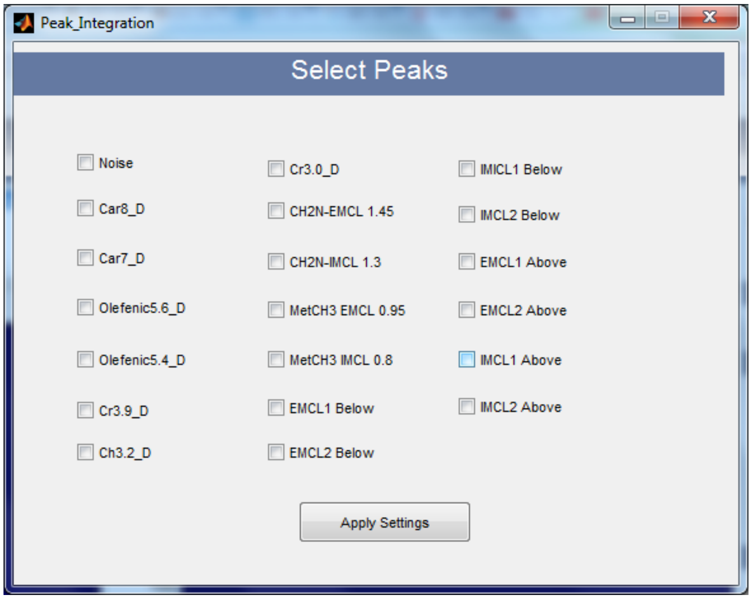

S3. A) The spectrum below was generated using the 2-Dimensional Plot option. B - The Multivoxel Settings window allows users to select specific metabolite peaks by setting the ranges (F1,F2 from 2-D plot) desired. The ensuing multivoxel plot will display the specified ranges for each voxel within the X-Y ranges set in the Multi-Voxel Display portion of the main window.

A)

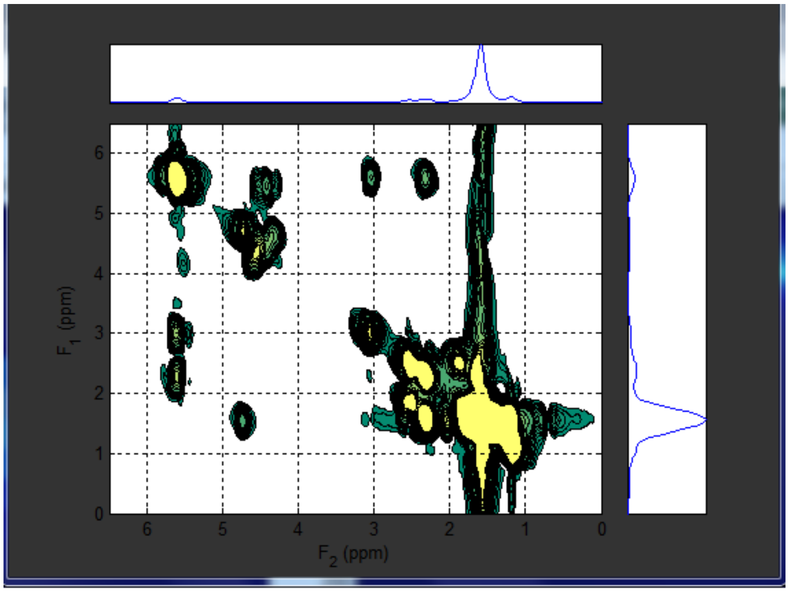

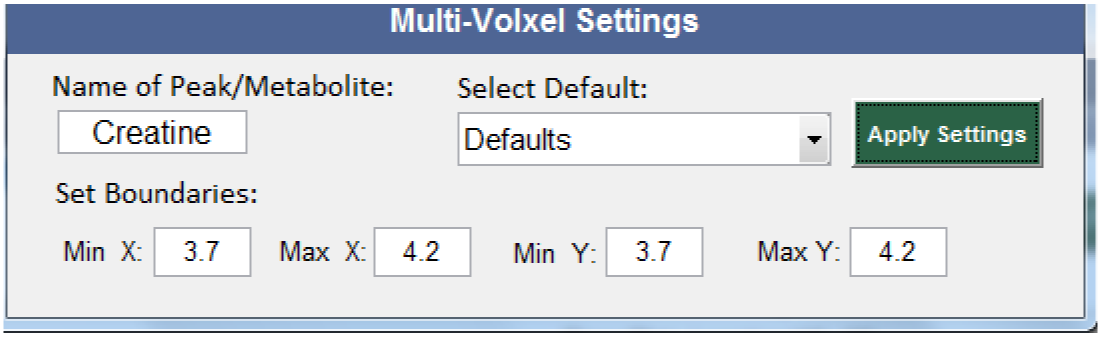

B)
